# Trichinœ

**Published:** 1866-07

**Authors:** 


*Trichinæ.*—The Medical Press and Circular states that in a case reported by Dr. Thudichum, (that of a German, 58 years of age,) he calculates the number of worms contained in the body of the patient, at about 40,000,000. A microscopic specimen of the flesh would frequently show upward of fifty of these capsules, and there were parts where the muscle seemed to consist of almost nothing but such capsules.

